# Supplementary material for: Xuebijing Administration Alleviates Pulmonary Endothelial Inflammation and Coagulation Dysregulation in the Early Phase of Sepsis in Rats
Source: J Clin Med. 2022 Nov 11;11(22):6696. doi: 10.3390/jcm11226696 (PMC9694218; doi:10.3390/jcm11226696)
Supplement: Supplementary file 1 [file jcm-11-06696-s001.zip › jcm-1987804-supplementary.pdf]

Supplemental Table S1. Mean arterial pressure within 12 h after cecal ligation and puncture in rats.

Groups	MAP (mmHg)												
	Before CLP	After CLP											
		1 h	2 h	3 h	4 h	5 h	6 h	7 h	8 h	9 h	10 h	11 h	12 h
Sham	104.0±5.2	102.7±14.3	92.6±18.8	96.0±5.6	88.4±19.5	102.0±14.9	99.0±14.8	86.7±3.5	84.5±1.53	79.4±3.5	83.0±11.9	91.0±8.2	83.4±5.5
CLP	94.7±7.1	96.5±4.2	95.6±5.5	90.7±10.2	81.0±11.6	81.6±6.8	77.7±10.9	81.0±15.0	73.0±7.5	61.6±6.0	63.8±5.0	58.7±7.0**	48.8±12.9**
CLP + xuebijing	94.0±12.5	83.1±11.8	90.3±14.2	88.0±11.4	94.6±14.0	90.7±19.3	91.6±15.3	82.0±8.5	66.0±5.2	74.3±9.5	65.3±8.1	58.7±8.3**	55.4±4.0$^{*\#}$

CLP, cecal ligation and puncture; $^{*}P< 0.05$, $^{**}P< 0.01$ vs. sham; $^{\#}P< 0.05$ vs. CLP group; n = 3.

Supplemental Table S2. Endothlin-1 plasma levels in rats.

Groups	Endothlin-1 plasma levels (pg/mL) after CLP				
	2 h	4 h	6 h	8 h	12 h
Sham	18.15±3.81	22.40±0.81	20.79±2.16	19.49±2.16	19.18±1.79
CLP	21.14±0.93	23.47±1.65	26.72±1.65*	29.02±1.92*	30.23±2.16*
CLP + xuebijing	23.14±3.92	21.13±1.14#	20.98±1.41	25.87±4.11#	22.28±2.24#

CLP, cecal ligation and puncture; *P< 0.05 vs. sham group; #P< 0.05 vs. CLP group; n = 5.

Supplemental Table S3. Von Willebrand factor plasma levels in rats.

Groups	Von Willebrand factor plasma levels (ng/dL) after CLP				
	2 h	4 h	6 h	8 h	12 h
Sham	6.06±0.87	5.06±0.98	4.09±0.87	2.99±0.98	2.48±0.56
CLP	5.21±1.67	5.69±1.41	6.64±1.78*	7.54±0.91*	9.13±0.97*
CLP + xuebijing	6.21±2.01	10.45±3.13#	9.51±2.52	5.66±1.45#	4.29±0.82#

CLP, cecal ligation and puncture; *P< 0.05 vs. sham group; #P< 0.05 vs. CLP group; n = 5.

Supplemental Table S4. Interleukin 6 plasma levels in rats.

Groups	Interleukin 6 plasma levels (pg/mL) after CLP				
	2 h	4 h	6 h	8 h	12 h
Sham	131.64±30.22	88.68±35.84	91.95±42.23	71.02±22.46	81.42±20.67
CLP	137.47±44.54	150.22±61.05	211.30±17.65*	283.52±39.55*	327.18±71.59*
CLP + xuebijing	120.83±33.91	94.54±12.59	112.12±19.58#	246.42±91.45	241.19±59.14

CLP, cecal ligation and puncture; **P*< 0.05 vs. sham group; #*P*< 0.05 vs. CLP group; n = 5.

Supplemental Table S5. Interleukin 1β plasma levels in rats.

Groups	Interleukin 1β plasma levels (pg/mL) after CLP				
	2 h	4 h	6 h	8 h	12 h
Sham	86.01±13.34	66.01±13.04	62.58±14.16	52.11±4.66	49.92±8.10
CLP	106.76±30.19	136.87±38.7*	160.39±28.2*	141.96±39.01*	1147.69±229.54*
CLP + xuebijing	54.73±14.35#	104.09±33.45	111.17±34.45	131.90±36.82	114.42±31.13#

CLP, cecal ligation and puncture; *P< 0.05 vs. sham group; #P< 0.05 vs. CLP group; n = 5.

Supplemental Table S6. Tumor necrosis factor-α plasma levels in rats.

Groups	Tumor necrosis factor-α plasma levels (pg/mL) after CLP				
	2 h	4 h	6 h	8 h	12 h
Sham	117.16±28.21	134.36±36.99	115.73±18.92	117.69±10.01	114.20±27.38
CLP	170.69±22.04*	291.66±74.31*	257.71±53.24*	208.84±13.51*	263.93±32.6*
CLP + xuebijing	129.86±21.77$^{\#}$	195.47±52.9$^{\#}$	158.18±35.69$^{\#}$	141.38±43.68$^{\#}$	165.73±43.24$^{\#}$

CLP, cecal ligation and puncture; $^{*}P< 0.05$ vs. sham group; $^{\#}P< 0.05$ vs. CLP group; n = 5.

Supplemental Table S7. Activated protein C plasma levels in rats.

Groups	Activated protein C levels (pg/mL) after CLP				
	2 h	4 h	6 h	8 h	12 h
Sham	2.133±0.784	1.713±0.371	2.445±0.434	2.704±0.676	3.418±0.440
CLP	1.694±0.639	0.632±0.146*	0.619±0.207*	0.319±0.214*	0.391±0.15*
CLP + xuebijing	2.573±0.897	3.782±0.844#	4.332±0.702#	2.698±0.935#	1.800±0.499#

CLP, cecal ligation and puncture; *$P < 0.05$ vs. sham group; #$P < 0.05$ vs. CLP group; n = 5.

Supplemental Table S8. Tissue plasminogen activator plasma levels in rats.

Groups	Tissue plasminogen activator levels (ng/mL) after CLP				
	2 h	4 h	6 h	8 h	12 h
Sham	1.705±0.507	1.233±0.694	1.439±0.622	1.619±0.870	0.854±0.222
CLP	20.131±2.950*	18.042±7.608*	3.718±1.911*	2.657±1.093	1.977±0.916*
CLP + xuebijing	2.688±0.471#	2.122±0.403#	6.168±0.793	10.396±1.763#	18.203±2.851#

CLP, cecal ligation and puncture; **P*< 0.05 vs. sham group; #*P*< 0.05 vs. CLP group; n = 5.
